# When Resilience Becomes Risk: A Latent Class Analysis of Psychosocial Resources and Allostatic Load Among African American Men

**DOI:** 10.1177/15579883221104272

**Published:** 2022-06-27

**Authors:** Courtney S. Thomas Tobin, Ángela Gutiérrez, Christy L. Erving, Keith C. Norris, Roland J. Thorpe

**Affiliations:** 1 Department of Community Health Sciences, Fielding School of Public Health, University of California, Los Angeles, Los Angeles, CA, USA; 2 Department of Social Medicine, Ohio University Heritage College of Osteopathic Medicine, Athens, OH, USA; 3 Department of Sociology, University of Texas at Austin, Austin, TX, USA; 4 Geffen School of Medicine, University of California, Los Angeles, Los Angeles, CA, USA; 5 Program for Research on Men’s Health, Johns Hopkins Bloomberg School of Public Health, Baltimore, MD, USA

**Keywords:** resilience, psychosocial resources, allostatic load, African American men, physiological health risk, latent class analysis, coping

## Abstract

There is a well-established link between psychosocial risks and psychological health among African American (AA) men. Yet, the psychosocial sources and physical health consequences of resilience (i.e., the ability to maintain good health despite adversity) remain underexplored. Using data from 283 AA men in the Nashville Stress and Health Study, the present study investigated the links between psychosocial resilience and allostatic load (AL), a biological indicator of physiological dysregulation. Latent class analysis (LCA) identified distinct resilience profiles comprising eight psychosocial resources across four categories: coping strategies, sense of control, racial identity, and social support. Analysis of variance (ANOVA) tests determined significant class differences in men’s AL scores. LCA results confirm a four-class model was the best fit: Class 1 (high resources, 32%), Class 2 (high coping but low control, 13%), Class 3 (low resources but high racial identity, 20%), and Class 4 (low resources but high mastery, 34%). Results reveal lower AL (better health) among Classes 1 (*m* = 0.35) and 4 (*m* = 0.31) and higher AL (worse health) among Classes 2 (*m* = 0.44) and 3 (*m* = 0.44). Findings indicate that the “quality” rather than the “quantity” of psychosocial resources matters for physical health among AA men, as positive health outcomes were observed among both low- and high-resource classes. Results suggest different resource combinations produce distinct patterns of resilience among AA men and underscore the need to further elucidate complex resilience processes among this population.

African American men experience disproportionately higher rates of chronic disease and accelerated declines in physical health status compared with other racial and gender groups in the United States (Chae et al., 2014; Thorpe, Bell, et al., 2015; Warner & Hayward, 2006). These disparities begin at younger ages and persist over time, resulting in shorter life expectancy and higher risk for premature mortality (Thorpe et al., 2013a, 2013b). Poor health outcomes among African American men have been attributed to structural inequality and elevated exposure to psychosocial stressors, many of which are the result of historical and contemporary racism (Griffith et al., 2013; Hargrove & Brown, 2015; Williams, 2003, 2015). For instance, due to racial residential segregation, African American men face greater lifetime exposure to environmental toxins, poor community conditions, underresourced schools, violence, incarceration, and food insecurity relative to White men (Sewell, 2016; Thorpe et al., 2019; Williams, 2003). These exposures may be frequent and chronic, shaping health through interactive processes that link psychosocial, behavioral, physiological, and biological factors across the life course (Geronimus et al., 2006; Griffith, 2015; Thorpe, Duru, & Hill, 2015; Thorpe et al., 2019).

African American men also face unique stressors due to the dual nature of their social position as members of a marginalized and minoritized racial group, while simultaneously experiencing gender-based privileges as men (T. H. Brown et al., 2016; Griffith, 2015; Hammond & Mattis, 2005). For instance, African American men report more experiences of discrimination than African American women (Navarrete et al., 2010; Pieterse & Carter, 2007). While discrimination exposure decreases with higher socioeconomic attainment among women, studies show a greater prevalence of discrimination among high socioeconomic status (SES) African American men (Griffith et al., 2011; Hudson et al., 2012). This suggests that African American men and women experience different manifestations of racial barriers (Griffith et al., 2011; Ifatunji & Harnois, 2016). More importantly, these patterns suggest race–gender disparities persist across SES levels and may constitute additional risk for men’s health trajectories (Thomas, 2015; Williams, 2003). Despite increased interest in gender as a fundamental determinant of health among men of color (Ellis et al., 2015; Griffith, 2012, 2015; Hargrove & T. H. Brown, 2015), we still know relatively little about the specific mechanisms through which gendered and racialized experiences translate into African American men’s health across the life course (Thorpe & Archibald, 2019).

Clark et al. (1999) developed a biopsychosocial model to ascertain the mechanisms through which racism, as a stressor, influences the health of African Americans. The present study makes use of this model as an orienting theoretical framework. Relatively understudied compared with the massive literature on racism and health, coping resources comprise a critical dimension of the model (Clark et al., 1999). Clark and colleagues (1999) recommended further research on racially influenced coping resources such as John Henryism, religiosity, and social support. In addition, they suggest that sociodemographic factors (e.g., age, educational attainment, and household income) might influence access to coping resources (Clark et al., 1999), a possibility that we consider here among African American men.

To better understand the ways that social and psychological experiences become embodied and contribute to inequalities among African American men, scholars have increasingly utilized biological indicators, such as allostatic load (AL), to assess social disparities in health (Geronimus et al., 2006; Green & Darity, 2010). AL refers to the cumulative wear and tear on the body’s physiological systems due to high-effort coping and adaptation to stressors (Geronimus et al., 2006; Turner et al., 2016). Specifically, when individuals perceive stressors, multiple physiological systems are activated as part of the body’s generalized stress response (Chyu & Upchurch, 2011; McEwen, 1998; McEwen & Seeman, 1999). Chronic, ongoing stress can diminish the body’s ability to effectively manage subsequent exposures (Karlamangla et al., 2006; McEwen & Seeman, 1999; Seeman et al., 2001). With its consideration of multiple physiological systems, AL provides a comprehensive assessment of physical health status compared with single-indicator health outcomes (McEwen & Seeman, 1999). AL also captures the impact of co-occurring chronic conditions and preclinical health status across multiple systems (T. N. Brown et al., 2016). Thus, AL is particularly useful for evaluating health among socially disadvantaged groups such as African American men, because it considers the long-term biological impact of repeated experiences with social adversity, economic disadvantage, and political marginalization, all of which may be linked to the early onset of chronic conditions among this group (Geronimus et al., 2006; Green & Darity, 2010; Thomas Tobin et al., 2019). While numerous studies have documented stark inequalities in AL across race and gender groups (Cobb et al., 2016; Geronimus et al., 2006; Thomas Tobin et al., 2019; Turner et al., 2017), fewer have focused on African American men (Thorpe et al., 2020). Consequently, the extent to which the unique social positioning and experiences of African American men as a racially minoritized group contribute to distinct physiological outcomes remains unclear.

This article highlights *psychosocial resilience* as a key, but understudied pathway linking the social and psychological contexts of African American men’s lives to their elevated risk of negative physiological outcomes. Most studies have focused on identifying risk mechanisms by assessing structural, behavioral, psychological, and biological risk factors that contribute to the disproportionately high burden of physical health risks among African American men (Gilbert et al., 2016; Thorpe et al., 2013a, 2020). Nevertheless, there is a growing interest in the protective factors that contribute to resilience. Resilience refers to an adaptive process that facilitates good health despite lifetime experiences of adversity (Abdou et al., 2010; Brody et al., 2016; Jackson et al., 2018). Social stress theory highlights the role of psychosocial resources and posits that these social and personal attributes help individuals navigate stressful events and buffer the negative health consequences of stress (Turner, 2013). Clark and colleagues’ (1999) biopsychosocial model further suggests that positive resources such as social support, mastery, racial identity, and effective coping styles may significantly contribute to resilience across the life course. Yet, there has been limited empirical consideration of the ways that psychosocial resources may contribute to resilience and shape physical health outcomes among African American men. These knowledge gaps undermine culturally tailored health promotion efforts for this population.

Prior research suggests that the availability of resilience-promoting psychosocial resources is less prevalent among individuals in disadvantaged social positions, such as African American men (Erving & Thomas, 2018; Thomas Tobin et al., 2021; Turner & Roszell, 1994). Those with more psychosocial resources at their disposal may be better equipped to successfully negotiate stressful situations, decreasing risk for poor health (Bonanno & Mancini, 2008; Pearlin et al., 2007; Turner & Roszell, 1994; Turner et al., 2004). Over time, individuals learn strategies that they perceive to be advantageous for reducing the psychological or physiological burden of stress (Bonanno & Mancini, 2008; Miller et al., 2011). It is through the process of appraising stressful events, utilizing available resources, evaluating their effectiveness, and adapting throughout the life course that individuals develop resilience, or the capacity to “bounce back” in the face of adversity (Windle, 2011). Thus, individuals are typically considered “resilient” if they possess a greater variety of psychosocial resources that facilitate their ability to maintain positive well-being despite experiencing a great deal of stress (Stewart & Yuen, 2011). Given that access to psychosocial resources varies across the life course (Pearlin et al., 2005), enhancing the availability of protective psychosocial resources that promote resilience may be an effective approach to reduce disparities and improve health among African American men.

Despite substantial conceptualization of resilience in prior research, there have been few empirical investigations of this construct and its linkages to health among African American men. Nevertheless, this small but growing body of research has documented the importance of psychosocial resources, such as John Henryism or active coping (James, 1994), sense of control (Watkins et al., 2011), spirituality and religiosity (Bowie et al., 2017; Bruce & Thorpe, 2019; Taylor et al., 2004), racial identity (Demo & Hughes, 1990; Mahalik et al., 2006), and social support (Mouzon, 2013, 2014; Nguyen et al., 2015) for enhancing well-being among African American men. Yet, this line of inquiry has yielded only a limited understanding of resilience. For instance, these studies generally investigate a single psychosocial resource, although prior research suggests that resilience is a dynamic process shaped by numerous psychosocial resources. As such, individual psychosocial resource dimensions would not capture the broad range of resources that collectively promote resilience. Given that prior research assessing the relationship between psychosocial resources and health has primarily focused on the role of individual resources, the potentially synergistic impact of multiple psychosocial resources has been overlooked, ultimately undermining the opportunity to identify the most effective combinations of resources that may promote resilience and improve health (Wilson et al., 2016).

Despite the growing recognition of the distinct stressors faced by African American men (T. H. Brown et al., 2016; Griffith et al., 2013; Hargrove & T. H. Brown, 2015; Thorpe et al., 2017; Williams, 2003, 2015), prior research on resilience has not considered psychosocial resources that might be unique to this population. For instance, gender-specific expectations and responsibilities for men, such as providing for one’s family, may diminish men’s sense of control; relatedly, these expectations may shape willingness to draw on social support to cope with major stressors, such as unemployment (Bowman, 1989; Griffith et al., 2011; Watkins et al., 2011). The combination of high stress exposure and limited psychosocial resources can contribute to diminished resilience and increased health risks for African American men. This notion is challenged by research arguing that resilience processes may simply operate differently among high-risk populations (Mezuk et al., 2013), whose psychosocial resources may be transformed by their gendered and racialized experiences (Pierre & Mahalik, 2005; Pierre et al., 2001). Thus, the lived experiences of African American men may give rise to distinct profiles of psychosocial resilience that influence their physical health trajectories.

The purpose of the present study was to identify the psychosocial resources that promote physiological resilience among African American men. First, we used latent class analysis (LCA) to identify distinct, multidimensional classes of psychosocial resources among African American men. This person-centered approach grouped individuals into latent classes based on their access to multiple resources, which allowed us to better understand patterns across the population. Second, we assessed the sociodemographic correlates associated with each latent class to determine whether certain subgroups of African American men were more likely to have access to particular resources. Finally, we evaluated the extent to which AL, a biomarker of cumulative physiological dysregulation, varied across latent classes. By investigating the distinct patterns and physiological impact of psychosocial resources among this group, this study aimed to enhance our understanding of the ways that psychosocial resources may promote resilience and favorable physical health outcomes among African American men.

## Method

The Nashville Stress and Health Study (NSAHS) is a population-based sample of African American and White adults aged 21 to 69 drawn from the city of Nashville and surrounding areas within Davidson County, Tennessee. A random sample was obtained using a multistage, stratified sampling approach. Although African American households were oversampled, sampling weights allowed for generalizability to the county population. Between 2011 and 2014, 1,252 respondents, including 297 African American men, provided information about their personal and family backgrounds, stress and coping experiences, and health histories during 3-hr computer-assisted interviews with interviewers of the same race. African Americans provided additional information about exposure to race-related stressors and racial group identity. Interviews included in-home clinician visits the following day. Clinicians arrived before breakfast to retrieve 12-hr urine samples and collect blood samples; measure blood pressure; take waist, hip, height, and weight measurements; and document prescription medication usage. Upon completion of the interviews, American Association for Public Opinion Research (AAPOR) rates were used to evaluate success across screening and interviewing phases (Response Rate 1 = 30.2, Cooperation Rate 1 = 74.2, Refusal Rate 1 = 30.2, Contact Rate 1 = 40.7). All participants provided informed consent. The NSAHS and all study procedures were approved by the Vanderbilt University Institutional Review Board and described in detail elsewhere (see T. N. Brown et al., 2016). Due to difficulty in drawing sufficient blood specimen contamination, or clinician visit refusal, less than 1% of the sample was missing sociodemographic or biological data. The present analyses included 283 African American men for whom complete data were available. Sample characteristics of the analytic sample are provided in Table 1.

**Table 1.** Sample Characteristics of African American Men, Nashville Stress and Health Study (2011–2014).

Characteristics	*N*	*M* or %	*SD*	Range
Allostatic load	283	0.37	0.27	0–1
Age	283	43.15	15.72	22–66
Education				
Less than High School (HS; Ref.)	62	19.54		
HS/GED	91	26.82		
Some college	77	32.20		
College or higher	53	21.43		
Annual household income <$20,000 (Ref.)	83	29.33		
$20,000–$34,999	53	18.73		
$35,000–54,999	59	20.85		
$55,000–$74,999	35	12.37		
$75,000–$94,999	25	8.83		
$95,000+	28	9.89		
Occupational prestige	283	40.96	29.53	0–100
Marital status				
Married (Ref.)	125	47.45		
Never married	82	32.58		
Other	76	19.97		

*Note.* Weighted means and percentages are presented. Ref. = reference category; *SD* = standard deviation.

### Measures

#### AL

Composite AL scores were derived from 10 biomarkers: (1) norepinephrine, (2) epinephrine, (3) cortisol, (4) dehydroepiandrosterone sulfate (DHEA-S), (5) total cholesterol, (6) high-density lipids (HDL), (7) glycated hemoglobin, (8) systolic blood pressure, (9) diastolic blood pressure, and (10) waist-to-hip ratio. Consistent with prior research (e.g., Geronimus et al., 2006), each biomarker was designated (0) low risk or (1) high risk based on established clinical risk levels (Turner et al., 2016); individuals taking blood pressure or cholesterol medication were also counted as “high risk” for those biomarkers. AL scores were based on the proportion of high-risk indicators for each respondent and ranged from 0 to 1, with higher values corresponding with higher levels of AL (T. N. Brown et al., 2016). Based on prior research, respondents with total scores consisting of 4 out of 10 high-risk biomarkers (or 0.40) were recognized as having “high AL,” consistent with elevated levels of physiological dysregulation (Geronimus et al., 2006; Thomas Tobin et al., 2019).

#### Psychosocial Resources

Eight psychosocial resources across four categories were examined: (1) coping strategies, (2) sense of control, (3) racial identity, and (4) social support.

##### Coping Strategies

Two types of coping were assessed: John Henryism and religious coping. *John Henryism* concerns one’s motivations to work hard and persevere through adversity (Bennett et al., 2004; James, 1994). A 12-item scale (e.g., “When things don’t go the way I want them to, that just makes me work even harder”; α = .74) was used, and responses were measured on a 5-point Likert-type scale ranging from 1 (*completely false*) to 5 (*completely true*). John Henryism scores were created by summing the items, such that higher values indicated greater use of high-effort, active coping strategies. *Religious coping* was assessed with a single item: “How often do you turn to your religion or spiritual beliefs to help you deal with daily problems?” Responses ranged from 1 (*never*) to 4 (*always*), with higher values indicating a greater tendency to use religious or spirituality-based coping strategies.

##### Sense of Control

Mastery and sense of divine control are two dimensions that capture the degree to which individuals feel that they are in control of their lives. *Mastery* assesses men’s general sense of efficacy in attaining goals and solving problems (Pearlin & Schooler, 1978). Respondents rated their agreement with 7 items (α = .71) including “You have little control over the things that happen to you.” Responses ranged from 1 (*strongly agree*) to 5 (*strongly disagree*) and were summed so that higher scores indicated higher mastery. *Divine control* was measured with a 4-item scale (α = .71) that evaluated the extent to which individuals feel their lives and outcomes are shaped by God and are a part of a divine plan (Schieman et al., 2005). Items included “I decide what to do without relying on God (reverse coded),” “When good or bad things happen, I see it as part of God’s plan for me,” “God has decided what my life shall be,” “I depend on God for help and guidance.” Responses categories ranged from 1 (*strongly disagree*) to 4 (*strongly agree*). Items were summed and higher scores indicate greater sense of divine control.

##### Racial Identity

The NSAHS assessed the extent to which African Americans identified with their racial heritage (Turner, 2013). Results from confirmatory factor analyses indicated two distinct constructs most closely aligned with “racial centrality” and “closeness to other Blacks” (hereafter referred to as “closeness to other Black people”; Demo & Hughes, 1990; Sellers et al., 1998). *Racial Centrality* evaluated the degree to which individuals feel being Black is central to their self-concept (Sellers et al., 1998). It was measured with 4 items (α = .75) including “You have a strong sense of yourself as a member of your racial/ethnic group.” *Closeness to Other Black People* was measured by 6 items (α = .78) that assessed the degree to which individuals feel connected to and share commonalities with other African Americans (e.g., “Your values, attitudes, and behaviors are shared by most members of your racial/ethnic group”; Demo & Hughes, 1990). Responses for both identity dimensions ranged from 1 (*strongly disagree*) to 7 (*strongly agree*). Items were summed so higher values corresponded with a greater sense of a central Black identity and feelings of closeness to other Black people.

##### Social Support

*Family support* measured individuals’ perceptions of being able to rely on family for emotional and instrumental support in times of need. Eight items such as “You feel very close to your family” and “No matter what happens you know that your family will always be there for you should you need them” were used. The same items were adapted to examine perceived *friend support* (Turner & Marino, 1994). Responses ranged from 1 (*not at all true for you*) to 4 (*very true for you*) and were summed so higher scores indicated greater support. Reliability was high for both family (α = .90) and friend (α = .94) support measures.

#### Sociodemographic Characteristics

*Age* was measured continuously in years. Respondents were between ages 18 and 69. *Education* was assessed categorically: less than high school, high school/GED, some college, and college graduate or higher. Respondents provided information about their *annual household income* (0 = $20,000; 1 = $20,000–$34,999; 2 = $35,000–$54,999; 3 = $55,000–$74,999; 4 = $75,000–$94,999; 5 = $95,000+) and *occupational prestige*, which evaluated individual social class standing based on the perceived prestige of their job position. Scores ranged from 0 to 100 based on the Nam–Powers–Boyd occupational status scale (see Turner et al., 2016), and higher scores corresponded with higher occupational prestige. Men’s *marital status* (0 = *married*; 1 = *never married*; 2 = *other*) was assessed.

### Statistical Analyses

**Figure 1.**
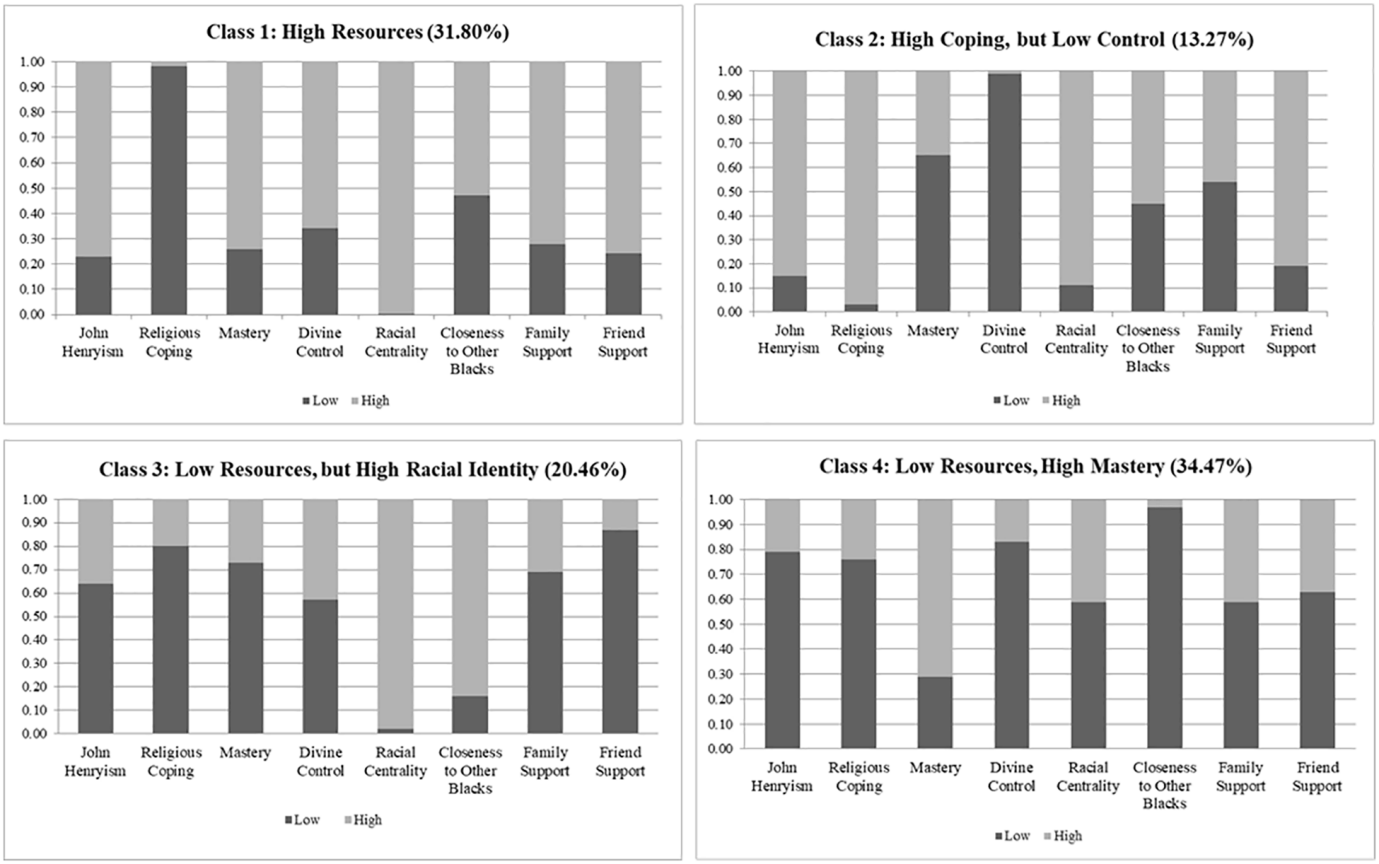
Probability of Item Endorsement Across Psychosocial Resources Latent Classes Among African American Men, Nashville Stress and Health Study (2011–2014; N = 283).

**Figure 2.**
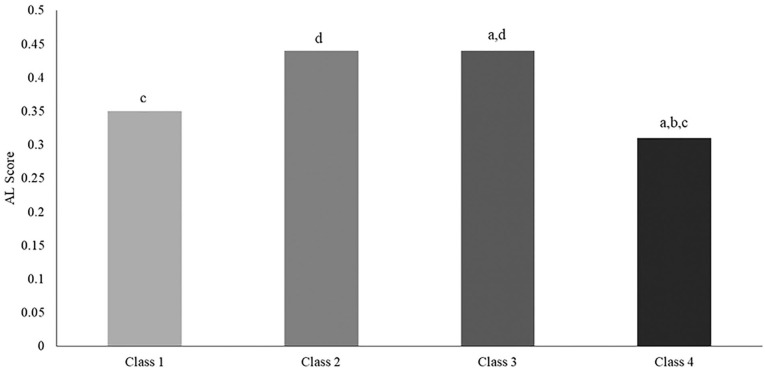
Mean Allostatic Load (AL) by Psychosocial Resource Latent Classes Among African American Men. *Note*. Data: Nashville Stress and Health Study (2011–2014; N = 283). Results indicate the following about each latent class: Class 1: n = 90, m = 0.35, SE = 0.04; Class 2: n = 37, m = 0.44, SE = 0.04; Class 3: n = 58, m = 0.44, SE = 0.03; Class 4: n = 98, m = 0.31, SE = 0.03. The letters above each bar indicate statistically significant differences at p < .05. (a) Denotes that the AL of members in this class is significantly different from the AL of Class 1 members; (b) indicates significant differences from Class 2; (c) indicates significant differences from Class 3; (d) indicates significant differences from Class 4.

Means, standard deviations, and frequencies were used to characterize the sample. The analytic approach was conducted by objective. To address the first objective, LCA was first used to identify patterns in the availability of psychosocial resources among African American men. This person-centered approach classifies respondents into subgroups (i.e., latent classes) based on their response patterns across multiple categorical indicators (Lincoln & Nguyen, 2021; McCutcheon, 1987; Nguyen, 2017). This method was preferred over variable-based models, which only examine relationships between an outcome and its predictors (Lincoln et al., 2007; Meiser & Ohrt, 1996). In the present study, latent classes described the co-occurrence of the eight psychosocial resources among African American men. The LCA included several steps. First, each resource score was dichotomized into “low” and “high” categories based on the median, to allow for comparability across different resources. Second, a model designating the number of latent classes that best fit the data was specified, and model fit and number of latent classes were evaluated (see Figure 1) using several parameters including the Bayesian information criterion (BIC), Akaike information criterion (AIC), likelihood ratio (LR) test, *G*^2^, and entropy. Smaller BIC, AIC, LR, and *G*^2^ values suggest better model fit. Entropy denotes the extent to which classes are clearly distinguishable from one another based on individuals’ probability of being in each class, and higher values are preferred (Muthén & Muthén, 2002). Figure 2 illustrates the probability of “low” or “high” endorsement for each psychosocial resource within each latent class. Table 2 presents the percentage of men endorsing low versus high levels of the resources within each latent class.

**Table 2.** Model Selection Criteria for Latent Class Analysis of Psychosocial Resources Among African American Men, Nashville Stress and Health Study (2011–2014; N = 283).

Selection criteria	Two latent classes	Three latent classes	Four latent classes	Five latent classes
Bayesian information criterion (BIC)	444.5	442.07	443.62	478.96
Akaike information criterion (AIC)	382.52	347.28	316.03	318.56
Likelihood ratio (LR)	−1,418.69	−1,392.1	−1,367.44	−1,359.71
*G* ^2^	348.52	295.28	246.03	230.56
Entropy	64.88	121.87	130.77	121.81

*Note.* Based on these selection criteria, a four-class latent model was selected. BIC = Bayesian information criterion; AIC = Akaike information criterion; LR = likelihood ratio.

For the second objective, unadjusted weighted means and percentages were estimated to assess sociodemographic variations across latent classes. Analysis of variance (ANOVA) tests were used for continuous predictors and χ^2^ tests were used for categorical predictors to determine whether these mean or proportional differences were statistically significant across latent classes. To address the third objective, the mean AL score of each latent class was estimated; significant differences (based on ANOVA tests) indicated class membership was associated with distinctions in the AL scores of African American men. All analyses controlled for age, education, income, occupational prestige, and marital status. All analyses were performed using STATA 15.1 software and used sampling weights to account for the complex multistage sampling design of the NSAHS sample.

## Results

Table 1 presents descriptive statistics for the sample. The average AL score was 0.37 (*SD* = 0.27), indicating that most men had nearly 4 high-risk biomarkers of the 10 total biomarkers assessed. The average age of men in the sample was 43 years old (*m* = 43.15; *SD* = 15.72); 26.82% (*n* = 91) were high school graduates, and 21.43% (*n* = 53) were college graduates. Most (68.91%, *n* = 195) earned less than $55,000 annually, with 20.85% (*n* = 59) earning $35,000 to $54,999, 18.73% earning $20,000 to $34,999 (*n* = 53), and 29.33% (*n* = 83) earning less than $20,000. The average occupational prestige score was 40.96 (*SD* = 29.53; e.g., technical/clerical positions). More than half were currently married (47.45%, *n* = 125) or formerly married (i.e., “Other”= Separated, widowed, divorced; 19.97%, *n* = 76). Approximately 32.58% (*n* = 82) were never married.

### LCA of Psychosocial Resources Among African American Men

Results of the LCA indicated that a four-class model was the best fit (see Table 2; BIC = 443.62; AIC = 316.03; entropy = 130.77; *G*^2^ = 246.03; LR = −1,367.44). Figure 1 displays the probability of item endorsement, which illustrates individuals’ likelihood of endorsing low versus high levels of resources within each latent class. Findings indicate that men in Class 1 (31.80%, *n* = 90) were more likely to endorse high levels of all psychosocial resources except religious coping, which was notably low in this group. Class 2 (13.27%, *n* = 37) was characterized by high levels of coping resources (i.e., John Henryism and religious coping), racial identity, and social support, but they were more likely to report low levels of control (i.e., mastery and divine control). While those in Class 3 (20.46%, *n* = 58) endorsed generally low resource levels, they were most likely to report high racial identity (i.e., racial centrality, closeness to other Black people) and equally likely to endorse low or high levels of divine control. Similarly, resource levels were generally low among Class 4 (34.47%, *n* = 98); this class of men were more likely to endorse high levels of personal mastery.

Table 3 presents significant differences in the proportions of each class that endorsed high versus low resources. For instance, 80.49% of Class 1 and 88.95% of Class 2 reported high levels of John Henryism compared with just 31.41% of Class 3 and 16.09% of Class 4 (*p* < .001). Significant class differences were observed across the other psychosocial resources as well, with one exception: Class differences in family support were not statistically significant. Taken together, these findings not only demonstrate that the availability of psychosocial resources varies significantly across latent classes of African American men, but also that the distribution of each resource is distinct and depends on class membership.

**Table 3.** Proportion of African American Men Endorsing Low Versus High Levels of Psychosocial Resources Across Latent Classes, Nashville Stress and Health Study (2011–2014; N = 283).

Psychosocial Resources	Class 1^a^ (*n* = 90)	Class 2 (*n* = 37)	Class 3 (*n* = 58)	Class 4 (*n* = 98)	*p* value
Coping strategies					
*John Henryism*					
Low	19.51	11.05	68.59	50.63	*p* < .001
High	80.49	88.95	31.41	16.09	
*Religious coping*					
Low	98.54	7.10	84.06	75.38	*p* < .001
High	1.46	99.29	15.94	24.62	
Sense of control					
*Personal mastery*					
Low	23.56	66.84	77.35	31.51	*p* < .01
High	76.44	33.16	22.65	68.49	
*Divine Control*					
Low	34.21	100.00	49.90	88.14	*p* < .001
High	65.79	0.00	50.10	11.86	
Racial identity					
*Racial centrality*					
Low	0.00	7.95	1.22	59.58	*p* < .001
High	100.00	92.05	98.78	40.42	
*Closeness to other Black people*					
Low	47.40	42.77	9.13	98.16	*p* < .001
High	52.60	57.23	90.87	1.84	
Social support					
*Family support*					
Low	33.19	51.87	67.74	57.18	*p* = .07
High	66.81	48.13	32.26	42.82	
*Friend support*					
Low	19.50	18.54	97.87	65.16	*p* < .001
High	80.50	81.46	2.13	34.84	

*Note.* Weighted proportions are presented.

a Class 1 is the reference category.

### Sociodemographic Correlates of Psychosocial Resource Classes

The sociodemographic factors associated with each latent class are examined in Table 4. Classes did not vary significantly in age or marital status. More men in Class 1 completed college (30.51%), had annual incomes of $75,000 to $94,999 (20.39%) and $95,000 (12.33%), and had higher occupational prestige scores (*m* = 45.54, *SE*=3.04; e.g., tax preparer, maintenance worker, customer service representative). In Class 2, 43.18% completed high school/GED, most earned $35,000 to $54,999 (57.55%), and the average occupational prestige score was 38.37 (*SE* = 4.32; e.g., actor, security guard, payroll clerk). Among men in Class 3, 37.35% had less than a high school education; most earned less than $20,000 (36.43%) or $20,000 to $34,999 (27.74%) annually and had an average occupational prestige score of 34.55 (*SE* = 2.58; e.g., mail clerk, retail sales agent, welder). In Class 4, 38.32% had some college experience and 21.16% were college graduates. Household income varied widely among men in this class, as 26.51% earned less than $20,000, 26.61% earned $35,000 to $54,999, and 13.65% earned $75,000-$94,999 each year; the average occupational prestige score in Class 4 was 41.55 (*SE* = 2.68; e.g., plumber, farmer, sheet metal worker). Overall, these patterns indicate there are significant sociodemographic differences across psychosocial resource latent classes among African American men.

**Table 4.** Sociodemographic Characteristics of Psychosocial Resource Classes Among African American Men, Nashville Stress and Health Study (2011–2014; N = 283).

Sociodemographic Characteristics	***Class 1*** 31.80%	***Class 2*** 13.27%	***Class 3*** 20.46%	***Class 4*** 34.47%
Age	41.19 (2.02)	43.74 (1.50)	45.89 (1.85)	43.10 (1.96)
Education				
Less than HS (Ref.)	15.47	22.70^c^	37.35^bd^	11.51^bc^
HS/ GED	18.61	43.18^c^	25.27^bd^	29.02^c^
Some college	35.40^c^	13.07^d^	29.34^a^	38.32^bcd^
College or higher	30.51^c^	21.05^c^	8.03^ab^	21.16
Annual household income <$20,000 (Ref.)	20.26^b^	25.1	36.43^b^	26.51
$20,000–$34,999	18.26^b^	8.33	27.74^b^	12.80
$35,000–54,999	14.05^b^	57.55^c^	20.63^b^	26.61
$55,000–$74,999	17.71^b^	6.44	10.21	15.80
$75,000–$94,999	20.39^bc^	1.74^acd^	0.00^ab^	13.65^bcd^
$95,000+	12.33^bc^	0.92^ac^	4.98^a^	5.92
Occupational prestige	45.54^b^ (3.04)	38.37^a^ (4.32)	34.55^a^ (2.58)	41.55 (2.68)
Marital status				
Married (Ref.)	46.67	39.64	44.24	53.08
Never married	36.42	36.96	35.51	25.61
Other	16.91	23.40	20.25	21.30

*Note.* Weighted, unadjusted means, and proportions are presented; standard errors are included in parentheses for continuous variables. Ref. = reference category.

Significant differences across latent classes (*p* < .05): Different from (a) Class 1, (b) Class 2, (c) Class 3, (d) Class 4.

### AL Across Psychosocial Resource Classes

Results identified significant differences in AL across psychosocial resource classes. Figure 2 depicts the age-adjusted mean AL of each latent class. Mean AL was lowest among Class 4 (*m* = 0.31, *SE*=0.03) and highest among Classes 2 and 3 (*m* = 0.44, *SE* = 0.03). There were no significant differences in AL scores between Class 1 (*m* = 0.35, *SE* = 0.04) and Class 4 (*m* = 0.31, *SE* = 0.03).

## Discussion

The overarching goal of this article was to shed new light on the ways that psychosocial resources may promote resilience and improve health among African American men. Drawing on Clark and colleagues’ (1999) biopsychosocial model, our three main objectives were to (1) use LCA to identify distinct, multidimensional classes of psychosocial resources among African American men; (2) assess the sociodemographic correlates associated with each latent class to determine whether certain subgroups of African American men were more likely to have access to particular resources; and (3) evaluate the extent to which AL varied across latent classes. Findings indicate that there are four distinct combinations of psychosocial resources among African American men and each of these classes is differentially associated with AL. Results underscore the importance of addressing psychosocial determinants of health and its connection to the accumulation of stress as indicated by AL.

Results revealed four distinct classes of psychosocial resources among African American men; these classes differ in regard to access to coping resources, sense of control, racial identity, and social support. Class 4, the most common class (34.47%), was characterized by low levels of most resources, yet high levels of mastery. Class 1, the second most common class (31.80%) consisted of African American men high in resources such as mastery, social support, and racial centrality, but with low levels of religious coping. All four classes revealed that each unique constellation of resources were not uniformly high or low; in fact, it is striking that each class is distinguishable with regard to having particularly high or low levels of a particular resource. These findings are consistent with some research confirming higher levels of individual psychosocial resources (e.g., social support and mastery) among African Americans compared with their White counterparts (Alang, 2014; Thomas Tobin et al., 2021). Yet, our results provide additional nuance for African American men in particular. The identification of four unique classes of psychosocial resources strongly suggests that resilience is not simply the sum total of resources, but rather a multidimensional collection of factors that likely have synergistic relationships with one another. In other words, it is the particular combination of resources, which vary in their content and levels, that constitute psychosocial resilience.

We identified sociodemographic differences across the four latent classes. Specifically, substantial SES differences across resource classes emerged. For instance, African American men in Class 3, characterized by low resources, yet salient racial identity, had relatively lower educational attainment and income vis-à-vis African American men belonging to Class 2 (high coping but low control). Class 1, characterized by high resources but low religious coping, contained the highest percentage of college-educated, high-income-earning men with relatively higher occupational prestige. This pattern is somewhat consistent with prior findings that socially advantaged groups have a greater availability of psychosocial resources (Kiecolt et al., 2009; Turner & Roszell, 1994). Greater socioeconomic attainment is linked to unique stress experiences for African American men (Bonham et al., 2004; Hudson et al., 2012; Thomas, 2015). Consequently, the types of psychosocial resources needed to resolve or counteract such stressors may vary by SES, resulting in similar, nonlinear patterns of resilience. Taken together, these findings demonstrate the complexities of psychosocial resilience among African American men and suggest they may be closely related to socioeconomic resources such as education, occupation, and income. The broader implication is that such SES differentials may contribute to differential resilience and health protectiveness among African American men. Stated differently, SES patterns of health among African American men may be in part attributable to differential access to resources. Additional research is needed to further evaluate potential relationships between SES, stress exposure, and psychosocial resilience among African American men.

As expected, psychosocial resilience was significantly associated with the AL of African American men in this study. These results diverge from previous research of resilience and mental health, which documents improved psychological functioning among African American men with greater availability of psychosocial resources (Wilson et al., 2016). For example, men in Classes 1 and 4 had similar AL scores, although Class 1 consisted of African American men with relatively high levels of resources (e.g., mastery, social support), whereas those in Class 4 were quite low in most resources (e.g., John Henryism, racial identity). It is especially noteworthy that Class 4, the group with the lowest psychosocial resources (with the exception of mastery), had relatively low AL compared with the other three classes, which comprised men who had relatively greater access to resources. The finding that men in Class 4 had similar AL levels as Class 1 members may point to the resilience process: that having comparatively lower AL among men with few psychosocial resources is a reflection of their resilience. It may be that psychosocial resources other than those assessed in the present study may contribute to resilience among this group and may protect against physiological dysregulation. For instance, prior research suggests that psychosocial factors, such as social cohesion, optimism, and self-affirmation, may promote resilience (Taylor & Broffman, 2011; Taylor & Seeman, 1999). Future research should assess a broad range of psychosocial factors that shape resilience among this population.

An alternative explanation for the similar AL levels between Class 1 and Class 4 may be related to diminishing returns in health. Prior research has documented diminishing returns in health for African Americans, such that African Americans and other racial and ethnic minoritized groups do not experience the same health benefits that are typically associated with higher SES (Bell et al., 2020; Braveman et al., 2010; Geronimus et al., 2006; Hudson et al., 2020). Findings from this study bolster prior research documenting diminishing returns in health among African Americans. For those in Class 1, having high levels of psychosocial resources and high educational, economic, and occupational prestige did not result in favorable health outcomes. Findings suggest that psychosocial resources and socioeconomic position alone do not fully protect against the adverse health consequences associated with distinct adversities African American men face, such as systemic racism and toxic masculinity. As such, future research should consider the role of both distal and proximal factors in contributing to physiological dysregulation among this population.

Interestingly, Class 2, characterized by high resources with low control, and Class 3, characterized by low resources with high racial identity, had *indistinguishable* AL scores. These patterns suggest that while some groups may have generally high resources (e.g., Class 2), if they lack access to particular resources—in their case, low levels of control—then they still face greater health risk. Similarly, groups with generally low resources but high mastery (e.g., Class 4) had the same health benefits as those with generally high resources (e.g., Class 1). Prior research among White Americans suggests that resources related to individuals’ sense of control, such as mastery, are the best candidates for improving mental health outcomes (Aneshensel, 2009; Turner & Lloyd, 1999). Findings from this study extend that line of research by identifying the unique role of control in shaping resilience and physical health among African American men. Collectively, findings demonstrate that it is the “quality” not “quantity” of resources that is consequential for physical health among African American men. The synergistic nature of psychosocial resources underscores the need to evaluate multiple psychosocial resources to better understand how they contribute to resilience and health promotion.

### Study Limitations and Future Research Directions

Results should be interpreted in the context of certain limitations. First, the cross-sectional design of the study precludes us from drawing causal inferences regarding the relationship between psychosocial resources and AL. Given that psychosocial resources vary over time (Taylor & Stanton, 2007), future longitudinal research should consider the ways that variations in psychosocial resources shape AL over time. Second, these data were collected on a sample of adults in an urban area in Tennessee. Future research should draw on nationally representative and longitudinal data to evaluate how these processes unfold over time. Nevertheless, in light of the limited availability of longitudinal and nationally representative data sources for African American men (Watkins et al., 2011), the NSAHS provides new insights, allowing for the consideration of a wide range of psychosocial resources and AL, a biomarker of physical health status among African American men. Third, while this study examined multiple psychosocial resources deemed important in prior research, there are several other dimensions that were not included in this analysis. For instance, there is a growing recognition of gendered processes for shaping the lived experiences and coping responses of African American men (Ellis et al., 2015; Hammond & Mattis, 2005). Dimensions such as racialized gender roles and the meanings attributed to being minority men may serve as resources that African American men use to confront adversity (Mahalik et al., 2006; Williams, 2003). Additional research is needed to identify the full range of psychosocial resources that African American men utilize throughout their lives and to pinpoint those most protective for physical health.

In addition to psychosocial resources, future research should consider the ways that racism shapes psychosocial resources and AL. Racism operates through distinct mechanisms, such as societal policies and norms, discrimination, and unfair treatment (Gee et al., 2019). A focus on the interactive relationships among racism, psychosocial resources, and physiological dysregulation aligns with Clark and colleagues’ (1999) biopsychosocial model and will enhance knowledge regarding the pathways through which both stress exposure and psychosocial resources jointly converge to influence the health of Black men. To mitigate persistent health disparities among African American men, future research should examine individual-, neighborhood-, and structural-level factors that shape psychosocial resources, resilience, and physiological dysregulation among African American men. Fourth, although this study recognized the essential role of exposure to social stressors in the development of psychosocial resilience over the life course, stress exposure was not directly assessed. Early life adversity may be an important mechanism through which resilience is developed and maintained over time (Thomas Tobin & Moody, 2021; Turner et al., 2016; Umberson et al., 2015). Additional research is needed to evaluate the significance of lifetime stress exposure for shaping African American men’s resilience.

### Contributions

This study extends our understanding of resilience and physiological dysregulation among African American men in several ways. First, we are among the first to examine resilience profiles among this population. We employed LCA, an integrative, person-centered approach, to identify multidimensional classes of psychosocial resources that shape physical health among African American men. We analyzed a broad range of psychosocial resources, including coping strategies, sense of control, racial identity, and social support. Ultimately, applying LCA to the study of resilience advances the research literature by assessing multiple psychosocial resources simultaneously rather than a single psychosocial resource at a time. Second, by drawing on Clark’s biopsychosocial model, we extend the research literature by documenting the ways through which combinations of psychosocial factors differentially contribute to physiological dysregulation among African American men. Very few studies have examined the correlates of AL among African American men specifically. By assessing AL, the present research sheds light on the cumulative physiological impact of these psychosocial resources. Findings advance current empirical evidence of integrative biopsychosocial health processes and highlight the need to evaluate the psychosocial mechanisms specific to African American men. Third, findings of the present study may inform culturally tailored health promotion programs. In addition to local and federal policies aimed at combating racism and its adverse health consequences, intervention programs should consider promoting resilience by enhancing a range of psychosocial resources among African American men. To improve the physical health trajectories of African American men, more research is needed to understand how psychosocial resilience develops over time, particularly in the context of social and environmental factors and psychosocial stressors.
